# PGAT-ABPp: harnessing protein language models and graph attention networks for antibacterial peptide identification with remarkable accuracy

**DOI:** 10.1093/bioinformatics/btae497

**Published:** 2024-08-09

**Authors:** Yuelei Hao, Xuyang Liu, Haohao Fu, Xueguang Shao, Wensheng Cai

**Affiliations:** Research Center for Analytical Sciences, Tianjin Key Laboratory of Biosensing and Molecular Recognition, State Key Laboratory of Medicinal Chemical Biology, College of Chemistry, Nankai University, Tianjin 300071, China; Haihe Laboratory of Sustainable Chemical Transformations, Tianjin 300192, China; Research Center for Analytical Sciences, Tianjin Key Laboratory of Biosensing and Molecular Recognition, State Key Laboratory of Medicinal Chemical Biology, College of Chemistry, Nankai University, Tianjin 300071, China; Haihe Laboratory of Sustainable Chemical Transformations, Tianjin 300192, China; Research Center for Analytical Sciences, Tianjin Key Laboratory of Biosensing and Molecular Recognition, State Key Laboratory of Medicinal Chemical Biology, College of Chemistry, Nankai University, Tianjin 300071, China; Haihe Laboratory of Sustainable Chemical Transformations, Tianjin 300192, China; Research Center for Analytical Sciences, Tianjin Key Laboratory of Biosensing and Molecular Recognition, State Key Laboratory of Medicinal Chemical Biology, College of Chemistry, Nankai University, Tianjin 300071, China; Haihe Laboratory of Sustainable Chemical Transformations, Tianjin 300192, China; Research Center for Analytical Sciences, Tianjin Key Laboratory of Biosensing and Molecular Recognition, State Key Laboratory of Medicinal Chemical Biology, College of Chemistry, Nankai University, Tianjin 300071, China; Haihe Laboratory of Sustainable Chemical Transformations, Tianjin 300192, China

## Abstract

**Motivation:**

The emergence of drug-resistant pathogens represents a formidable challenge to global health. Using computational methods to identify the antibacterial peptides (ABPs), an alternative antimicrobial agent, has demonstrated advantages in further drug design studies. Most of the current approaches, however, rely on handcrafted features and underutilize structural information, which may affect prediction performance.

**Results:**

To present an ultra-accurate model for ABP identification, we propose a novel deep learning approach, PGAT-ABPp. PGAT-ABPp leverages structures predicted by AlphaFold2 and a pretrained protein language model, ProtT5-XL-U50 (ProtT5), to construct graphs. Then the graph attention network (GAT) is adopted to learn global discriminative features from the graphs. PGAT-ABPp outperforms the other fourteen state-of-the-art models in terms of accuracy, F1-score and Matthews Correlation Coefficient on the independent test dataset. The results show that ProtT5 has significant advantages in the identification of ABPs and the introduction of spatial information further improves the prediction performance of the model. The interpretability analysis of key residues in known active ABPs further underscores the superiority of PGAT-ABPp.

**Availability and implementation:**

The datasets and source codes for the PGAT-ABPp model are available at https://github.com/moonseter/PGAT-ABPp/.

## Graphical abstract

**Figure:**
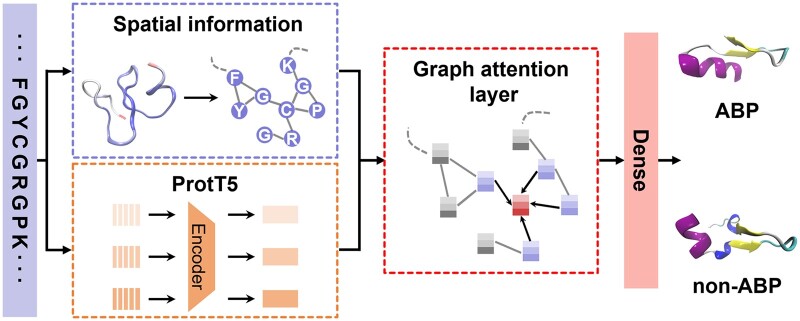

## 1 Introduction

The excessive utilization of antibiotics has led to the development of antibiotic resistance in nearly all documented bacterial pathogens (Li *et al.* 2023). This resistance continues to spread not only among the pathogenic bacteria of human and animal origins, but also among the environmental microorganisms (Sharma *et al.* 2014, Hendriksen *et al.* 2019). The dissemination of drug-resistant bacteria worldwide represents an escalating challenge to global public health (Roca *et al.* 2015, Raphael and Riley 2017). Antimicrobial peptides (AMPs), also known as host defense peptides, constitute a pivotal part of the immune defense system of organisms. AMPs exhibit multifaceted mechanisms of action and have broad-spectrum antibacterial activity against bacteria, fungi, viruses, and other pathogens (Andersson *et al.* 2016, Mookherjee *et al.* 2020, Hancock *et al.* 2021). Consequently, they have emerged as potential alternatives to conventional antibacterial drugs.

The identification of AMPs in wet laboratories requires intricate designs, long screening cycles and strict conditions, making large-scale screening very challenging (Hancock *et al.* 2021). Computer-assisted methods represent a crucial approach for the identification and design of AMPs due to their potential for large-scale preliminary screening prior to clinical trials. In the past few decades, many excellent machine learning-based methods have been developed for the identification of AMPs (Agrawal *et al.* 2021, Zhang *et al.* 2022, Zhou *et al.* 2022, Teimouri *et al.* 2023). Employing the support vector machine (SVM) method, Zhang *et al.* (2022) constructed an antifungal peptide classification model based on pre-calculated and filtered peptide descriptors. Moreover, they further utilized the support vector regression (SVR) to develop activity prediction models targeting four specified target fungi based on activity values. Aiming to develop a bacterium-specific machine-learning approach, Teimouri *et al.* (2023) used the least absolute shrinkage and selection operator (LASSO) regression and the SVM to select the most important physicochemical characteristics among 1537 descriptors generated by the propy package for each peptide. The test results obtained by Teimouri and the colleagues indicated that there is a distinct set of features related to antimicrobial activity for each bacterium, while some characteristics like the secondary structures are important for more than one bacterium.

Although many feature selection methods have been developed (Bolón-Canedo and Alonso-Betanzos 2019, Speiser *et al.* 2019, Anowar *et al.* 2021), handcrafted features, which can also be referred to as method-specific features, may omit crucial information pertaining to essential antimicrobial characteristics, thereby affecting the accuracy of identification. Protein sequences in 1D order are essentially similar to natural language in that amino acids are arranged in a variety of combinations to form functional structures, just as letters make up words and sentences that have meanings. Therefore, a substantial influx of deep learning-based natural language processing methods has permeated the field of protein research (Hu *et al.* 2022, Singh *et al.* 2022a, Fang *et al.* 2023a). Protein language models (PLMs), such as ESM (Rives *et al.* 2021), ProtTrans (Elnaggar *et al.* 2021), and xTrimoPGLM (Chen *et al.* 2024), pretrained on large-scale protein sequence databases, are recognized for the ability to comprehensively describe protein properties. ProtTrans, including models like ProtT5-XL-U50 and ProtBert-BFD, possesses the capability to extract fundamental physicochemical characteristics of amino acids, such as charge, polarity, and hydrophobicity. Furthermore, ProtTrans can acquire comprehensive insights into global constraints related to protein structure and function, thereby achieving outstanding performance in per-residue prediction of protein secondary structure, even in the absence of evolutionary information (Elnaggar *et al.* 2021).

PLMs have also been applied in the identification of AMPs (Cao *et al.* 2023, Du *et al.* 2023, Fang *et al.* 2023b). UniDL4BioPep (Du *et al.* 2023) combined ESM-2 and convolutional neural network (CNN) to train across 20 datasets containing 18 different bioactivities. ESM-2, an evolution of the original ESM, was utilized for peptide embeddings. UniDL4BioPep outperformed the previous models trained and evaluated on the same datasets on 15 of these datasets, demonstrating the superiority of PLMs in capturing peptide residue and positional information. Fang *et al.* (2023b) proposed a multi-view feature learning scheme that utilized a co-attention mechanism to integrate information derived from the PLM with evolutionary information and physicochemical properties separately, aiming to predict antifungal peptides (AFPs). The results indicated that the features extracted by the PLM contributed more significantly to predicting AFPs. However, such methods do not take advantage of the spatial information of peptides, which plays a crucial role in determining their properties and mechanisms of action (Zhao *et al.* 2021, Szymczak *et al.* 2023, Yan *et al.* 2023). Combining PLMs with peptide 3D structural information is a very promising direction for achieving more accurate predictions of peptide properties, which constitutes the primary thrust of the present work.

Graph neural network (GNN) can leverage the spatial information of biomolecules and performs well in solving various biological problems (Wu *et al.* 2023, Yan *et al.* 2023, An *et al.* 2024a,b, Wong *et al.* 2024). Graph attention network (GAT) (Veličković *et al.* 2018) is a widely utilized GNN method that incorporates the attention mechanism into the information propagation process and employs the multi-head attention mechanism (Vaswani *et al.* 2017) to stabilize the learning process. It can effectively capture the structural and other information across the entire graph, thereby improving the model performance on graph-level tasks. Moreover, analyzing the learned attention weights enables comprehensive utilization of the information carried by the peptide structure and provides new insights for the design of new natural product analogs (Ciulla and Kumar 2018, Veličković *et al.* 2018).

In this study, we introduce the PLM and GAT into the task aiming at identifying antibacterial peptides (ABPs), and propose an ABP predictor coined PGAT-ABPp. ABPs represent a significant subset of AMPs, with their antibacterial activity being a focal point of current research (Li *et al.* 2023). Our approach employs the state-of-the-art PLM, ProtT5-XL-U50 (ProtT5), to generate peptide embeddings and leverages GAT to capture discriminative features from both spatial information and peptide embeddings. Due to the lack of experimentally confirmed 3D structures, we utilize AlphaFold2 (Jumper *et al.* 2021) to predict the structures of all the peptides in the dataset for its excellent performance on predicting 3D atomic coordinates of protein (Kryshtafovych *et al.* 2021). The results show that PGAT-ABPp can identify ABPs with highly comparable accuracy and exhibit robust performance with strong generalization capabilities. Visual analysis of attention weights offers insights and guidance for further experimental exploration.

## 2 Materials and methods

### 2.1 Dataset

The main dataset (*S*) utilized for training, testing, and fine-tuning the model, as well as the independent test dataset (*S^IN^*) employed for unbiased comparisons, are sourced from the previous Deep-ABPpred (Sharma *et al.* 2021) work. Peptides with antibacterial activity are considered as ABPs regardless of its target bacterium, while peptides with no known antimicrobial activity are labeled as non-ABPs. *S* includes 1635 ABPs and 1485 non-ABPs, while *S^IN^* comprises 4017 ABPs and 5799 non-ABPs. In addition, there is no overlap between *S* and *S^IN^*. As not all sequences possess confirmed 3D structures in the same experimental condition, we utilize the ColabFold (Mirdita *et al.* 2022) implementation of AlphaFold2 to predict the structures of all the sequences in the dataset. Subsequently, the predicted structures were selected to constitute our dataset.

**Figure 1.**
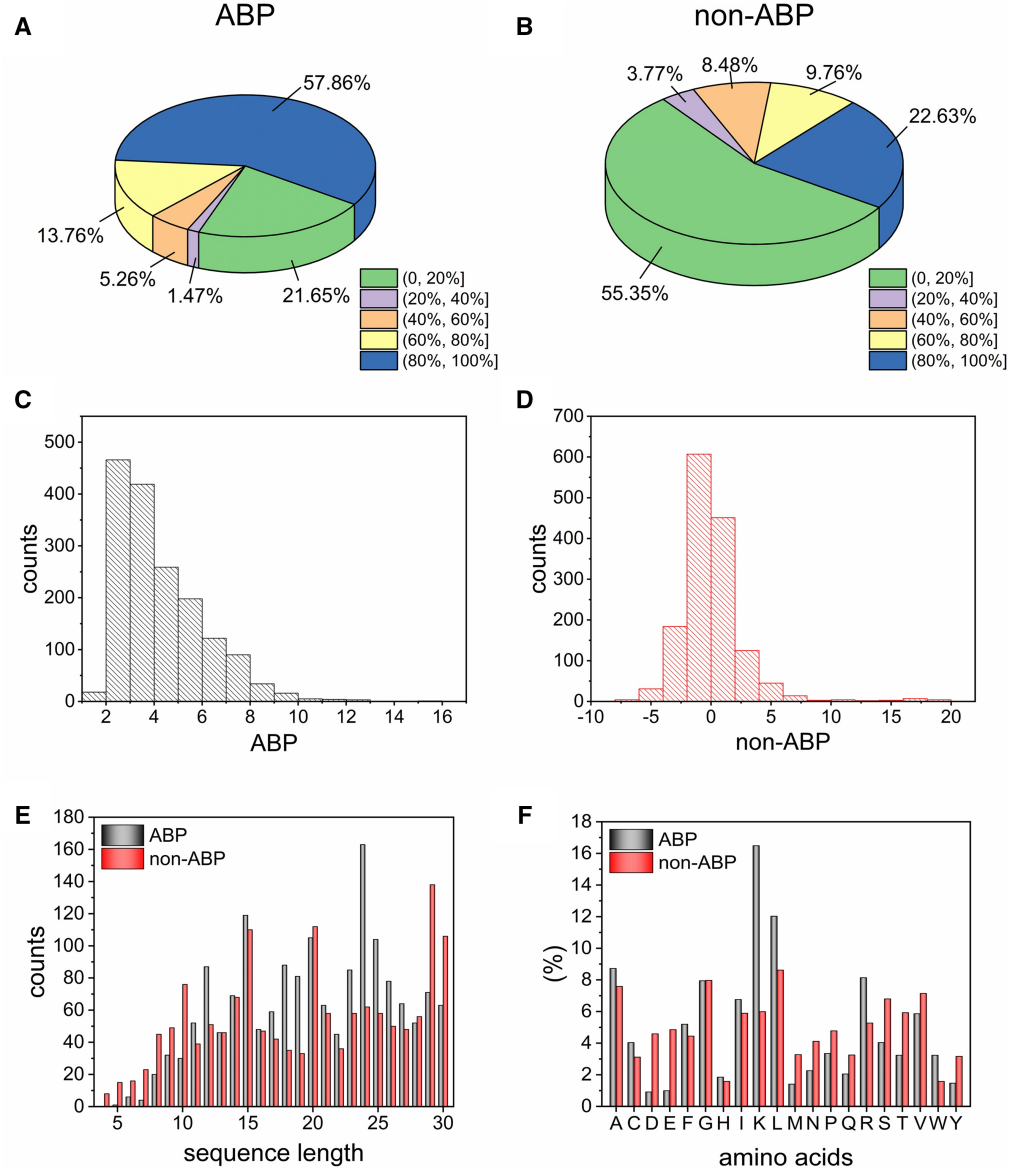
Statistics of the main dataset. Proportions of peptides with different α-helix contents within (A) ABPs and (B) non-ABPs. Different colors represent different α-helix content ranges, and the percentage of each sector in the pie chart represents the proportion of peptides within the corresponding range relative to all the peptides being analyzed. Charge distribution histogram of (C) ABPs and (D) non-ABPs. (E) Sequence lengths distribution of ABPs and non-ABPs. (F) Distribution of amino acids in ABPs and non-ABPs.

Statistics of the main dataset are shown in Fig. 1, and statistics of the independent test dataset are supplied in Supplementary Fig. S1. As illustrated in Fig. 1A and B, the proportion of peptides with high α-helix content in ABPs is notably higher than that of non-ABPs, whereas the proportion of peptides with low α-helix content in ABPs is correspondingly lower compared to non-ABPs. These differences in structures between ABPs and non-ABPs necessitate to be captured and used for distinguishing ABPs from non-ABPs. It can be seen that ABPs in the dataset possess higher net positive charge, whereas non-ABPs do not (Fig. 1C and D). According to the sequence length, ABPs are mainly distributed in the range of 15–25, while non-ABPs are primarily distributed in the range of 15–30 (Fig. 1E). In addition, Fig. 1F and Supplementary Fig. S1F reveal that ABPs are enriched with basic amino acids such as lysine and arginine, as well as hydrophobic amino acids such as alanine and leucine, in significantly higher abundance than non-ABPs. This enrichment is attributed to the requirement of ABPs to carry positive charge to form strong electrostatic interactions with bacterial cell membrane during initial binding, while hydrophobic residues interact with lipids to destroy the bacterial cell membrane (Brogden 2005, Gan *et al.* 2021).

### 2.2 Overview of PGAT-ABPp

**Figure 2.**
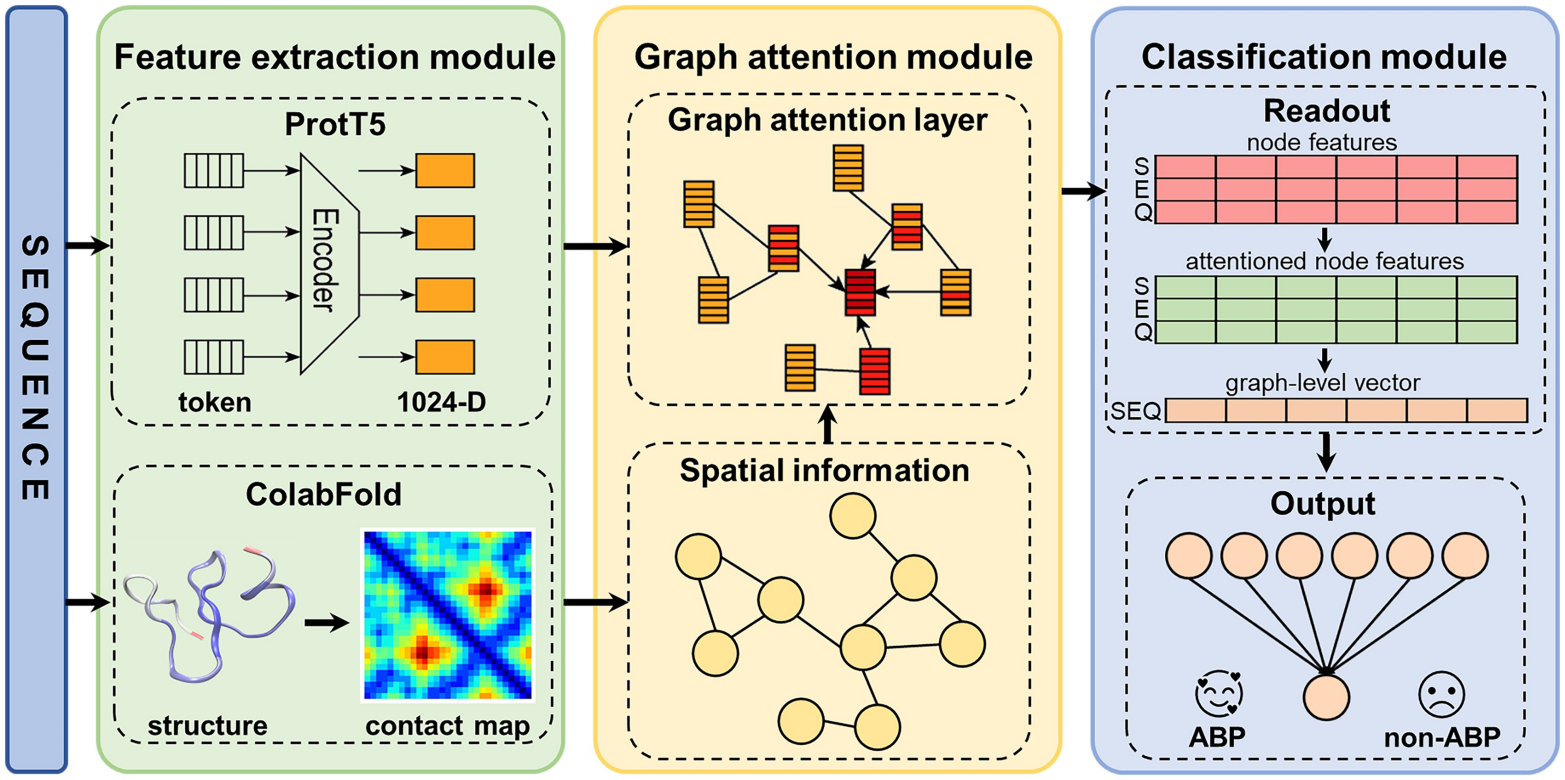
Framework of PGAT-ABPp. ColabFold predicts the 3D structure, from which the contact map is further generated. ProtT5 extracts node embeddings. The extracted spatial information and node embeddings construct the graph. The graph attention layer updates the node representations. The updated node features are then processed by the readout layer using an additional attention mechanism (resulting in the “attentioned node features”) and pooling technology to extract the graph-level representation as a single vector (“graph-level vector”). The output layer makes the final prediction for the sequence based on the readout vector.

As depicted in Fig. 2, PGAT-ABPp extracts spatial information from the predicted structure and combines it with the node embeddings extracted by ProtT5 to construct a graph. In the graph, nodes represent residue information, while edges represent the positional relationships between residues. Subsequently, we employ GAT to learn and update the node representations, which are then processed by the readout layer. Finally, the output layer is utilized to figure out whether the input is an ABP or not.

### 2.3 Pretrained protein language model

We choose ProtT5-XL-U50 (ProtT5) as the feature extractor to obtain node embeddings due to its ability to efficiently capture biophysical features of amino acids and protein structure classes, which are the properties we want to use for distinguishing ABPs and non-ABPs (Elnaggar *et al.* 2021). Moreover, ProtT5 is particularly well-suited for small families, making it ideal for ABPs (Elnaggar *et al.* 2021). ProtT5 adopts a 24-layer transformer architecture with 1024 hidden layers size, pretrained on the Big Fantastic Database (BFD) (Steinegger and Söding 2018, Steinegger *et al.* 2019) and subsequently fine-tuned on UniRef50 (Suzek *et al.* 2015). ProtT5 utilizes its encoder to generate context-aware embeddings for each input token. Given an input sequence *P* = (*p_1_*, *p_2_*, …, *p_n_*), where *n* denotes the sequence length, ProtT5 ultimately produces an embedding matrix with the shape of *n *×* *1024:

(1) H=ProtT5Encoder(P)

where ProtT5Encoder represents the pretrained ProtT5 without fine-tuning.

### 2.4 Graph attention module

#### 2.4.1 Graph representation

From the predicted structures, we can obtain the Cartesian coordinates for all atoms to create the contact maps. In this study, the distance between C_α_ atoms in the contact map is used to represent the spatial information. In that case, two residues are considered to be in contact if the distance between their corresponding C_α_ atoms falls within a certain range. The adjacency matrix, $A\in R^{n\times n}$, is then constructed, where n represents the number of amino acids in the sequence. The $A_{ij}$ is defined as follows (Gligorijević *et al.* 2021):

(2) $$A_{ij}=\{\begin{array}{cc} 1, & D_{ij}<D_{th}\\ 0, & \;\text{otherwise}\; \end{array}$$

where $D_{ij}$ is the distance between atoms *i* and *j*, and $D_{th}$ is the threshold distance, which is set to 10 Å in this study. For more information about the optimization, see Supplementary Fig. S2.

The graph is defined as $G=\left(V,E\right)$, where *V* and *E* represent the set of nodes and edges, respectively. $V=\left\{v_{i}\right\}$, where $v_{i}\in R^{1024}$ denotes the features of each node at the residue level. $E=\left\{e_{ij}|A_{ij}=1\right\}$ is determined by the adjacency matrix.

#### 2.4.2 Graph attention layer

GAT (Veličković *et al.* 2018) is utilized to learn structural information as well as graph-level information from the graph constructed above. The network is implemented through Keras (https://keras.io/) and TensorFlow (https://www.tensorflow.org/).

The input of graph attention layer is a set of node features, $H=\left\{\vec{h_{1}},\vec{h_{2}},\ldots ,\vec{h_{N}}\right\}$, where $\vec{h_{i}}\in R^{F}$ is the features of node *i*, as constructed above. Here, *N* denotes the number of nodes, and *F* represents the dimensionality of each node features. The self-attention mechanism is performed on each node to calculate attention coefficients that represent the importance of node *j* to node *i* (Vaswani *et al.* 2017, Veličković *et al.* 2018):

(3) $$\alpha_{ij}=\frac{\;\text{exp}\;\left(\mathrm{LeakyReLU}\left({\vec{a}}^{T}\left[W\vec{h_{i}}∥W\vec{h_{j}}\right]\right)\right)}{\sum_{k\in N_{i}}\;\text{exp}\;\left(\mathrm{LeakyReLU}\left({\vec{a}}^{T}\left[W\vec{h_{i}}∥W\vec{h_{j}}\right]\right)\right)}$$

where $W\in R^{F^{\prime }\times F}$ is utilized to apply a linear transformation for input node states, *F’* represents the dimensionality of the updated node features, $\vec{a}\in R^{2F^{\prime }}$ is a learnable weight vector, *T* represents transposition, ‖ represents concatenation. In this step, the masked attention mechanism is adopted, meaning that for each node *i*, only node $j\in N_{i}$ will be calculated, where $N_{i}$ represents a certain neighborhood of node *i* in the graph.

The normalized attention coefficients are used to calculate the linear combination of the corresponding features to obtain the output features of each node (Vaswani *et al.* 2017, Veličković *et al.* 2018):

(4) $${\vec{h_{i}}}^{\prime }=\sum_{j\in N_{i}}\alpha_{ij}W\vec{h_{j}}$$

To stabilize the learning process and enhance the generalization ability of the model, the multi-head attention mechanism is adopted. *K* independent attention mechanisms transform the node features according to eq 4, then their features are concatenated to obtain the output feature representation (Vaswani *et al.* 2017, Veličković *et al.* 2018):

(5) $${\vec{h_{i}}}^{\prime }=\overset{K}{\underset{k=1}{\left|\right|}}\left(\sum_{j\in N_{i}}\alpha_{ij}^{k}W^{k}\vec{h_{j}}\right)$$

where ‖ represents concatenation, $\alpha_{ij}^{k}$ represents the normalized attention coefficients obtained by the *k*th attention mechanism, and $W^{k}$ denotes the corresponding weight matrix used for linearly transforming the input.

### 2.5 Classification module

#### 2.5.1 Readout

As shown in Readout module of Fig. 2, the multi-head attention mechanism (Vaswani *et al.* 2017) is used to further handle the node features, providing a comprehensive view of node contexts within the graph. Subsequently, global average pooling (Lin *et al.* 2014) is utilized to compute the average of the multi-head outputs, resulting in a fixed-length graph-level feature vector x.

#### 2.5.2 Output

The read feature vector x is input into the dense layer, and the output is finally mapped to a range between 0 and 1 through the sigmoid activation function to identify the category of the input peptide:

(6) $$y=\frac{\;\text{exp}(x)}{1+\text{exp}(x)}$$

A peptide with a predicted *y* > 0.5 is considered as ABP, otherwise it is considered as non-ABP.

### 2.6 Evaluation metrics

In this study, we utilize six metrics to evaluate the model performance: accuracy (Acc), precision (Pr), specificity (Sp), the area under the receiver-operating characteristic curve (AUC), F1-score (Fs), and Matthews Correlation Coefficient (MCC). They are calculated by the following equations:

(7) $$\mathrm{Acc}=\frac{TP+TN}{TP+TN+FP+FN}$$

(8) $$Pr=\frac{TP}{TP+FP}$$

(9) $$Sp=\frac{TN}{TN+FP}$$

(10) $$Fs=\frac{2\times Pr\times Sn}{Sn+Pr}$$

(11) $$\mathrm{MCC}=\frac{\left(TP\times TN)-(FP\times FN\right)}{\sqrt{\left(TP+FP)\times (TP+FN)\times (TN+FP)\times (TN+FN\right)}}$$

where *TP*, *TN*, *FP*, and *FN* represent the number of true positives, true negatives, false positives, and false negatives, respectively.

The model implementation information is provided in Supplementary Material.

## 3 Results

### 3.1 Performance evaluation on the independent test dataset

In this section, we tested PGAT-ABPp on the independent test dataset, and compared it with fourteen state-of-the-art methods, including IAMPE-KNN (Kavousi *et al.* 2020), IAMPE-RF (Kavousi *et al.* 2020), IAMPE-SVM (Kavousi *et al.* 2020), IAMPE-XGBOOST (Kavousi *et al.* 2020), AMP Scanner vr.2 (Veltri *et al.* 2018), iAMPpred (Meher *et al.* 2017), ADAM (Lee *et al.* 2015), Deep-ABPpred (Sharma *et al.* 2021), amPEPpy (Lawrence *et al.* 2021), AMPDLMD (Cao *et al.* 2023), UniDL4BioPep (Du *et al.* 2023), sAMPpred-GAT (Yan *et al.* 2023), AMPpred-MFA (Li *et al.* 2024), and KNIME-best fused-feature model (Martínez-Mauricio *et al.* 2024). Both shallow models and deep models are included in these methods, while sAMPpred-GAT is considered to be the first model that utilizes predicted structures for AMP prediction. The results are summarized in Table 1. All models were trained on the same main dataset. PGAT-ABPp achieves the best performance on Acc, Pr, Sp, Fs, AUC, and MCC, ensuring efficient and accurate identification of active ABP instances during subsequent screening. Moreover, the statistical analysis results indicate that PGAT-ABPp exhibits significant advantages over amPEPpy, AMPDLMD, UniDL4BioPep, sAMPpred-GAT, AMPpred-MFA, as well as Deep-ABPpred in terms of Acc, Fs, and MCC (Supplementary Fig. S3).

**Table 1.** Performance of PGAT-ABPp and other methods on the independent test dataset.^a^

Methods	Acc^d^ (%)	Pr (%)	Sp (%)	AUC	Fs^d^	MCC^d^	Source
IAMPE-KNN	76.56	65.40	66.75	0.7874	0.7601	0.5706	Sharma *et al.* (2021)
IAMPE-RF	77.27	64.80	63.40	0.8034	0.7779	0.6130	Sharma *et al.* (2021)
IAMPE-SVM	65.94	54.66	43.49	0.7092	0.7027	0.4669	Sharma *et al.* (2021)
IAMPE-XGBOOST	74.51	61.90	58.19	0.7812	0.7589	0.5792	Sharma *et al.* (2021)
AMP Scanner vr.2	84.59	75.24	78.82	0.9308	0.8315	0.7056	Sharma *et al.* (2021)
iAMPpred	79.97	69.66	72.71	0.8922	0.7870	0.6223	Sharma *et al.* (2021)
ADAM	70.54	58.32	51.38	0.7479	0.7318	0.5268	Sharma *et al.* (2021)
Deep-ABPpred	95.80	93.67	95.49	0.9908	0.9494	0.9138	Sharma *et al.* (2021)
amPEPpy^b^	92.42	88.32	91.40	0.9817	0.9102	0.8459	Lawrence *et al.* (2021)
AMPDLMD^b^	93.71	90.80	93.32	0.6479	0.9246	0.8711	Cao *et al.* (2023)
UniDL4BioPep^b^	92.55	91.09	88.11	0.9796	0.9125	0.8498	Du *et al.* (2023)
sAMPpred-GAT^b^	88.95	82.60	86.41	0.9647	0.8730	0.7916	Yan *et al.*(2023)
AMPpred-MFA^b^	93.66	88.36	90.96	0.9896	0.9268	0.8754	Li *et al.* (2024)
KNIME-best fused-feature model	95.33	–	96.02	–	–	0.9035	Martínez-Mauricio *et al.* (2024)
PGAT-ABPp^c^	**96.49 ± 0.21**	**95.31 ± 0.51**	**96.72 ± 0.38**	**0.9936 ± 0.0007**	**0.9573 ± 0.0025**	**0.9280 ± 0.0042**	This study

a Note: “–” denotes the result is not available from the original papers, and the best performance of each metric is marked in bold.

b Results for models were obtained by training and evaluating them on our datasets. The given average values were obtained after performing the randomness initialization parameters 10 times.

c Results of the proposed model are the average and standard deviation obtained after performing the randomness initialization parameters 10 times.

d Statistical analysis was conducted using one-way ANOVA, *p*_Acc_*, p*_Fs_*, p*_MCC_ < 0.0001. More results in Supplementary Fig. S3.

### 3.2 10-Fold cross-validation on the main dataset

The 10-fold cross-validation method was employed to assess the robustness of our model using the main dataset. By executing 10-fold cross-validation, we acquired performance metrics of the model on different data subsets, enabling a comprehensive evaluation of its performance under varied conditions. The results, presented in Table 2, show that both the Fs and MCC of the model maintain high values, which are consistent with the results on the independent test dataset. Moreover, minimal fluctuations observed in the metrics suggest that our model is robust and suitable for real-world applications.

**Table 2.** 10-Fold cross-validation results of PGAT-ABPp.

Fold number	Acc (%)	Pr (%)	Sp (%)	AUC	Fs	MCC
1	97.76	97.75	97.04	0.9909	0.9803	0.9543
2	96.47	96.10	96.23	0.9862	0.9642	0.9295
3	98.08	99.99	99.99	0.9930	0.9813	0.9622
4	96.47	98.19	97.87	0.9871	0.9674	0.9295
5	98.08	99.37	99.33	0.9857	0.9814	0.9618
6	96.47	96.91	96.64	0.9900	0.9662	0.9294
7	97.12	98.66	98.73	0.9822	0.9703	0.9427
8	96.47	96.45	95.83	0.9848	0.9674	0.9291
9	98.08	99.99	99.99	0.9939	0.9817	0.9622
10	96.79	97.40	97.45	0.9887	0.9677	0.9360
mean	97.18 ± 0.74	98.08 ± 1.41	97.91 ± 1.53	0.9882 ± 0.0037	0.9728 ± 0.0074	0.9437 ± 0.0149

### 3.3 Ablation experiments

To assess the effectiveness of ProtT5 and the significance of incorporating structural information, we conducted ablation experiments. Previous studies categorized feature encoding methods into two major categories: peptide-level features and amino acid-level features (Sharma *et al.* 2021, Singh *et al.* 2022b, Yan *et al.* 2022). To evaluate the impact of different kinds of node features on the results, we selected the one-hot encoding method to obtain sequence-based features and the word2vec (Mikolov *et al.* 2013) method to obtain amino acid-level features. Accordingly, the models were named Onehot-GAT and Word2vec-GAT, respectively. Given the superior performance of CNN in ProtTrans downstream tasks (Elnaggar *et al.* 2021, Du *et al.* 2023), we used ProtT5-CNN to compare its performance with ProtT5-GAT, aiming to observe the significance of structural information.

**Figure 3.**
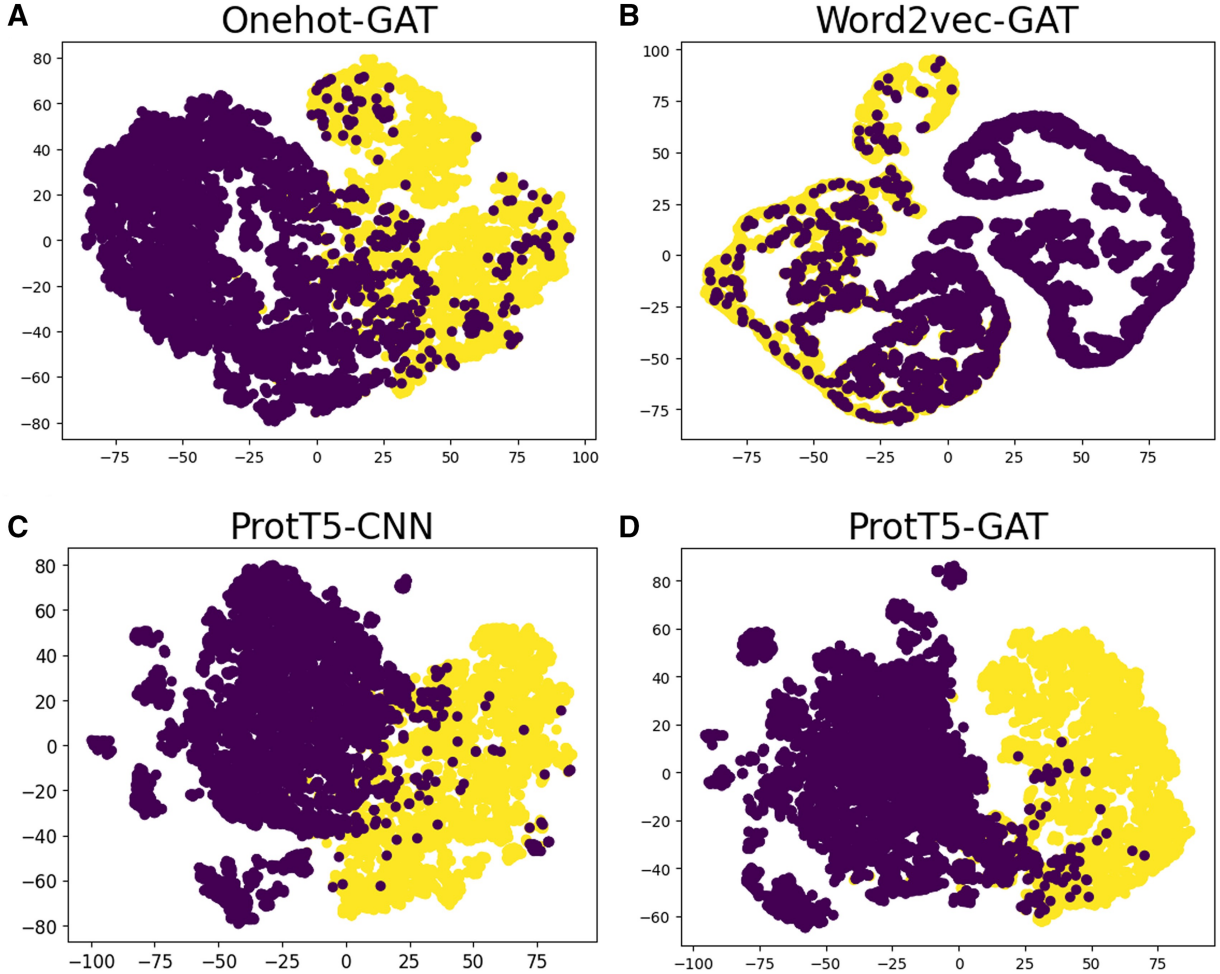
T-SNE results of (A) Onehot-GAT, (B) Word2vec-GAT, (C) ProtT5-CNN, and (D) ProtT5-GAT (our model). The purple (dark) dots represent non-ABPs, while the yellow (light) dots represent ABPs.

The results presented in Table 3 demonstrate the superior performance of ProtT5-GAT across various indicators. Compared to one-hot encoding and word2vec, the performance of the model significantly improves when using ProtT5 as the feature extractor. Specifically, compared to Onehot-GAT, the performance of ProtT5-GAT exhibits enhancements of 4.34%, 0.0479, and 0.0835 in Acc, Fs, and MCC, respectively, indicating that ProtT5 has a significant advantage in representing peptide sequences. Moreover, in comparison to ProtT5-CNN, the performance of ProtT5-GAT shows improvements of 0.88%, 0.0093, and 0.0170 in Acc, Fs, and MCC, respectively. Notably, ProtT5-GAT demonstrates a lower probability of misidentifying non-ABP as ABP, which may be attributed to the spatial structure learned by GAT (Fig. 3 and Supplementary Fig. S4). Furthermore, it is notable that higher number of graph attention layers does not always bring better performance (Supplementary Table S1).

**Table 3.** Performance of Onehot-GAT, Word2vec-GAT, ProtT5-CNN and ProtT5-GAT (our model) on ablation experiments on the independent test dataset.^a^^,^^b^

Methods	Acc (%)	Pr (%)	Sp (%)	AUC	Fs	MCC
Onehot-GAT	92.15 ± 0.90	87.33 ± 3.90	89.44 ± 1.43	0.9776 ± 0.0039	0.9094 ± 0.0098	0.8445 ± 0.0170
Word2vec-GAT	88.25 ± 3.26	79.83 ± 5.17	82.85 ± 5.62	0.9623 ± 0.0142	0.8710 ± 0.0308	0.7775 ± 0.0539
ProtT5-CNN	95.61 ± 0.16	92.30 ± 0.36	94.37 ± 0.28	0.9924 ± 0.0005	0.9478 ± 0.0018	0.9110 ± 0.0031
ProtT5-GAT	**96.49 ± 0.21**	**95.31 ± 0.51**	**96.72 ± 0.38**	**0.9936 ± 0.0007**	**0.9573 ± 0.0025**	**0.9280 ± 0.0042**

a Results of the models are the averages and standard deviations obtained after performing the randomness initialization parameters 10 times.

b Note: Best performance of each metric is marked in bold.

### 3.4 Model interpretation

To explore the capacity of PGAT-ABPp in learning biological information, we visualized the importance of model features based on attention weights. Specifically, we selected four peptides, PGLa (GMASKAGAIAGKIAKVALKAL) (Zasloff 1987, Soravia *et al.* 1988), Hepcidin-25 (DTHFPICIFCCGCCHRSKCGMCCKT) (Aschi *et al.* 2010, Uelker *et al.* 2016), HNP-1 (ACYCRIPACIAGERRYGTCIYQGRLWAFCC) (White *et al.* 1995, Xie *et al.* 2022), and Magainin-2 (GIGKFLHSAKKFGKAFVGEIMNS) (Zasloff 1987, Hasan *et al.* 2018, Mourtada *et al.* 2019), which are known to have antibacterial activity, for attention weights visualization.

**Figure 4.**
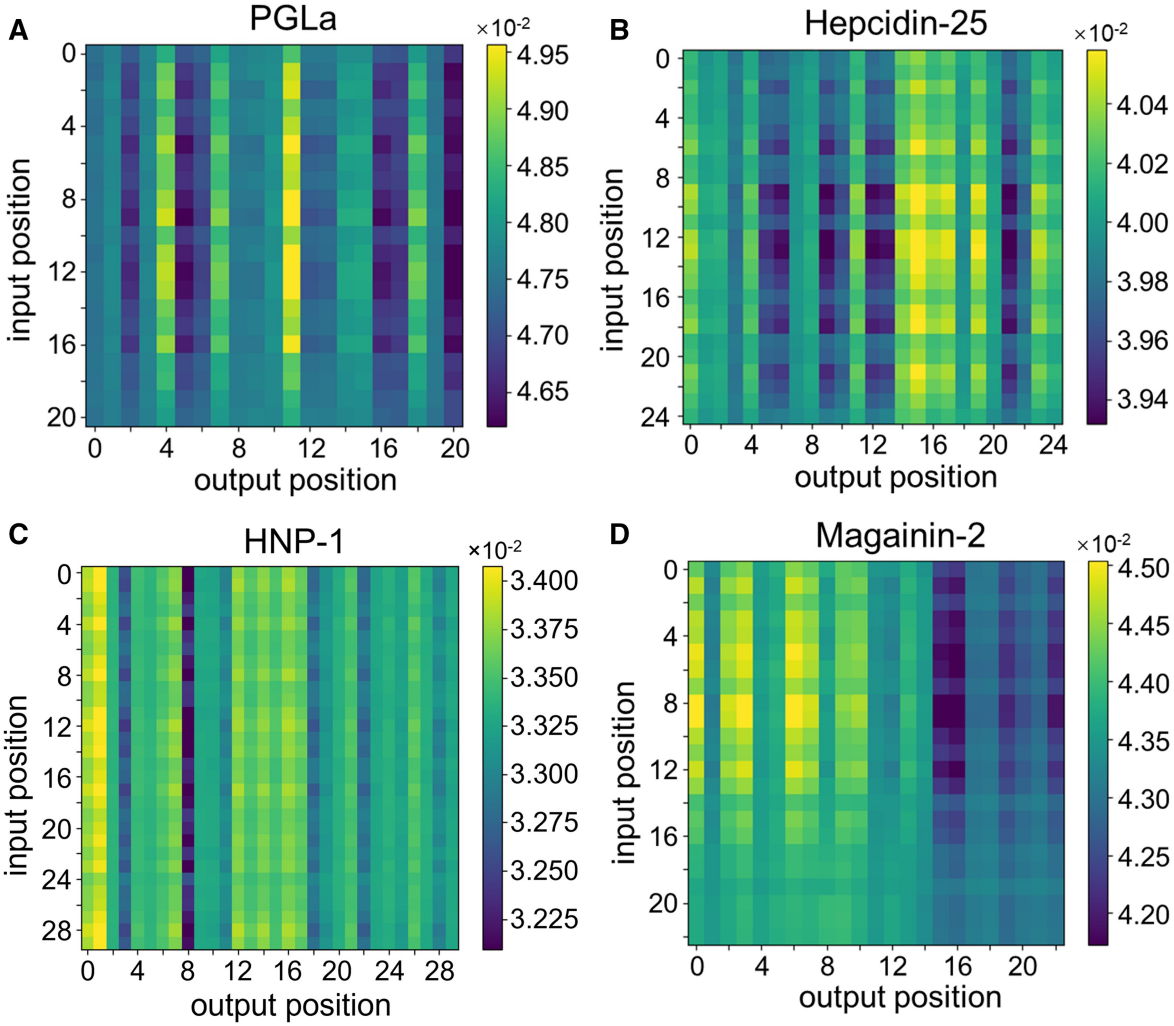
Attention weight heatmaps of (A) PGLa, (B) Hepcidin-25, (C) HNP-1, and (D) Magainin-2. The position index starting from 0 corresponds to residue number index starting from 1.

PGLa, found in frog skin, exhibits an unstructured form in aqueous solution but forms amphiphilic α-helices in membranes (Latal *et al.* 1997, Glaser *et al.* 2004). Bowers *et al.* (2022) revealed through molecular dynamics simulations that the strongest interaction between PGLa and DMPC/DMPG bilayer comes from the contact between Lys5 and DMPG phosphorus. They claimed that the free energy of PGLa binding to the bilayer is mainly determined by the balance between the desolvation of positive charges and electrostatic PGLa-lipid interactions. Moreover, the C-terminal α-helix undergoes rotation to maintain contact between lysines and anionic lipid phosphorus. These critical residues are assigned higher attention weights, which can be found at positions 4, 11, 14, and 18 in Fig. 4A, indicating the presence of positively charged lysines in PGLa. In addition, positions 7 and 15 correspond to hydrophobic amino acids, alanine and valine, respectively. The identified residues collectively contribute significantly to the antimicrobial property of PGLa.

In Hepcidin-25, the residues located at positions 14–19 in Fig. 4B exhibit higher weights, potentially attributed to their location in the turn regions of the β-sheet (Aschi *et al.* 2010). The heatmap analysis for HNP-1 (Fig. 4C) shows that the important N-terminus and sites with higher homology receive elevated higher weights, and the significant sites including positively charged and hydrophobic residues associated with the amphipathic nature of the peptide are identified (White *et al.* 1995). PGAT-ABPp recognizes all positively charged amino acids of Magainin-2 (positions 3, 6, 9, 10, and 13 in Fig. 4D). In addition, glycines located at positions 0 and 2, as well as serine located at position 7, which are important to maintain the antimicrobial conformation of Magainin-2, are also given higher attention weights (Mourtada *et al.* 2019).

## 4 Discussion

PGAT-ABPp is a robust and accurate ABP identification model. Given that the identification of ABPs serves as a preliminary screening for subsequent design or wet experiments, achieving higher accuracy is not only a metric evaluating model performance but also represents a promising threshold for further design. Ablation experiments demonstrate the advantages of protein language model over traditional feature embeddings in peptide embeddings tasks. The combination of features extracted by ProtT5 and spatial information of peptides shows great superiority in learning characteristics related to the antibacterial activity. The identification of essential residues of ABPs with different antibacterial mechanisms reflects the data sensitivity and generalization ability of PGAT-ABPp, proving its application potential in practical tasks and providing insights for mechanistic studies of ABPs. This capability bears significant implications for designing experimental strategies and guiding the direction of future research.

Although PGAT-ABPp shows superiority over other approaches, it still could be extended in several ways. First, in PGAT-ABPp, we utilized ProtT5 to extract peptide embeddings, indicating that the choice of PLMs affects performance and might be task-specific. Given the rapid progress in AI, more specialized biological PLMs will appear, potentially enhancing the performance of models. We employed predicted structures in this study, an innovative approach that achieved superior results, although it takes more time to prepare than using sequences directly (additional computational costs are detailed in the Supplementary Material). However, it is important to note that the configuration of ABPs in water might not be the same as that in membranes, so identifications using structures simulated in membranes may potentially be more accurate, although obtaining such structures presents significant challenges.

## 5 Conclusion

For the ultra-accurate identification of ABPs, we utilize the state-of-the-art PLM, ProtT5, along with peptide structures predicted by ColabFold to represent peptides, and propose a novel model named PGAT-ABPp. The PGAT-ABPp framework leverages the GAT network to learn the inherent features related to antibacterial activity from structural information and embeddings generated by ProtT5. PGAT-ABPp is a robust model for accurately identifying ABPs and exhibits notable advantages over other state-of-the-art models. The incorporation of GAT not only enables comprehensive use of structural information, but also brings interpretability benefits.

In summary, developing identification models for ABPs with high accuracy remains an essential task. The exceptional performance of PGAT-ABPp makes it a promising tool for the subsequent discovery and design of ABPs.

## Supplementary Material

btae497_Supplementary_Data
