# Supplementary material for: PGAT-ABPp: harnessing protein language models and graph attention networks for antibacterial peptide identification with remarkable accuracy
Source: Bioinformatics. 2024 Aug 9;40(8):btae497. doi: 10.1093/bioinformatics/btae497 (PMC11338452; doi:10.1093/bioinformatics/btae497)
Supplement: btae497_Supplementary_Data [file btae497_supplementary_data.docx]

PGAT-ABPp: Harnessing Protein Language Models and Graph Attention Networks for Antibacterial Peptide Identification with Remarkable Accuracy

Supplementary Material

Yuelei Hao^1,2^, Xuyang Liu^1,2^, Haohao Fu^1,2^, Xueguang Shao,^1,2,^* and Wensheng Cai^1,2,^*

^1^ Research Center for Analytical Sciences, Tianjin Key Laboratory of Biosensing and Molecular Recognition, State Key Laboratory of Medicinal Chemical Biology, College of Chemistry, Nankai University, Tianjin 300071, China

^2^ Haihe Laboratory of Sustainable Chemical Transformations, Tianjin 300192, China

**Corresponding Author**

**Wensheng Cai**

*E-mail: wscai@nankai.edu.cn

**Xueguang Shao**

*E-mail: xshao@nankai.edu.cn

[Supplementary methods 3](#_Toc170585451)

[ColabFold 3](#_Toc170585452)

[Dataset analysis 3](#_Toc170585453)

[ProtT5-XL-U50 3](#_Toc170585454)

[Evaluation metrics 4](#_Toc170585455)

[One-way ANOVA 5](#_Toc170585456)

[10-fold cross-validation 5](#_Toc170585457)

[One-hot encoding 5](#_Toc170585458)

[Word2vec 5](#_Toc170585459)

[t-SNE 6](#_Toc170585460)

[Model implementation and computational costs 6](#_Toc170585461)

[References 7](#_Toc170585462)

[Supplementary Figures and Tables 8](#_Toc170585463)

[Figure S1. 8](#_Toc170585464)

[Figure S2. 9](#_Toc170585465)

[Figure S3. 10](#_Toc170585466)

[Figure S4. 11](#_Toc170585467)

[Table S1. 12](#_Toc170585468)

Supplementary methods

# **ColabFold**

ColabFold is a free and accessible platform for protein folding. Researchers combine the fast homology search of MMseqs2 (Many-against-Many sequence searching) with AlphaFold2, providing a faster protein structure prediction software with performance comparable to AlphaFold2 (Mirdita *et al.* 2022). Using the Jupyter Notebook inside Google Colaboratory, users who lack the resources to run AlphaFold2 can get access ColabFold more easily. ColabFold first conducts an MMseqs2-based homology search server to build diverse MSAs (multiple sequence alignments) and to find templates. It then prepares features for AlphaFold2 to perform structure inference based on the MSA results. There are four main Jupyter Notebooks provided and LocalColabFold runs on users’ machines is also available. More information can be found online: https://github.com/sokrypton/ColabFold.

# **Dataset analysis**

The characteristics of sequences in the datasets, including peptide length, amino acid composition and net charge, were calculated using the Python library NumPy (https://numpy.org/) and modlAMP (https://modlamp.org/). The secondary structure analysis was conducted using VMD software (https://www.ks.uiuc.edu/Research/vmd/).

# **ProtT5-XL-U50**

ProtT5-XL-U50 (ProtT5) is a pretrained protein language model that belongs to the ProtTrans family of models (Elnaggar *et al.* 2021). ProtT5 was trained using 8-way model parallelism with 3 Billion (3B) parameters. It was trained on BFD100 and fine-tuned on UniRef50. ProtT5 employs a transformer-based architecture, specifically an extension of the T5 model (Text-to-Text Transfer Transformer), which has been adapted for sequence translation. The encoder projects the input protein sequences to an embedding space, and the decoder generates an output based on the embedding of the encoder. The encoder is used to derive features (embeddings) for protein sequences. First, a protein sequence is tokenized and positional encoding is added, resulting a vector. The ProtT5Encoder then processes the vector to create context-aware embeddings for each input token. The last hidden state of the Transformer’s attention stack is extracted as protein features.

The configuration for pretraining ProtT5 is listed:

| Hyperparameter | ProtT5-XL | |
| --- | --- | --- |
| dataset | BFD100 | UniRef50 |
| number of layers | 24 | |
| hidden layers size | 1024 | |
| hidden layers intermediate size | 16384 | |
| number of heads | 32 | |
| dropout | 0.1 | |
| target length | 512 | |
| masking probability | 15% | |
| local batch size | 4 | 8 |
| global batch size | 4096 | 2048 |
| optimizer | AdaFactor | |
| learning rate | 0.01 | |
| training steps | 1.2M | 991K |
| warm-up steps | 10K | |
| number of parameters | 3B | |

# **Evaluation metrics**

In this study, we utilize six metrics to evaluate the model performance: accuracy (Acc), precision (Pr), specificity (Sp), the area under the receiver-operating characteristic curve (AUC), F1-score (Fs) and Matthews correlation coefficient (MCC). Acc is the ratio of correctly predicted instances to the total number of instances, indicating the overall correctness of the model. Pr, also known as positive predictive value, measures the ratio of accurate positive predictions out of all the positive predictions. Sp, also known as the true negative rate, represents the correct prediction ratio of negative instances. AUC represents the area under the ROC curve, which plots the true positive rate (sensitivity) against the false positive rate (1 - specificity). Fs is the harmonic mean of precision and recall, providing a balance between the two. MCC is a correlation coefficient between the observed and predicted binary classifications, ranging from -1 to +1. Fs and MCC are both regarded as balanced measures even for imbalanced datasets. In the manuscript, we take Fs and MCC as the main metrics. These metrics are calculated relying on the sklearn.metrics module of Scikit-learn Python library (https://scikit-learn.org/).

# **One-way ANOVA**

One-way analysis of variance (ANOVA) is a statistical technique used to determine whether there are any statistically significant differences between the means of three or more independent (unrelated) groups (Janczyk and Pfister 2023). A small *p* value (typically ≤ 0.05) indicates that at least one group mean differs from the others, but it does not specify which ones. In the context of one-way ANOVA, when significant differences are found, a post-hoc test is performed as a subsequent step to identify which specific group pairs differ by conducting pairwise comparisons between group means. In this study, Tamhane's T2 test was chosen for post-hoc analyses due to the presence of heterogeneity of variance. SPSS software (https://www.ibm.com/spss) was used to conduct one-way ANOVA and to explain the differences between models based on the metrics of Acc, Fs and MCC.

# **10-fold cross-validation**

The dataset was divided into 10 mutually exclusive subsets. Each subset was used as validation set, and the model was trained on the remaining nine subsets. By executing this procedure 10 times, ensuring that each subset served as a validation set exactly once, performance metrics of the model on different data subsets were acquired, enabling a comprehensive evaluation of its performance under varied conditions.

# **One-hot encoding**

One-hot encoding method is a basic and popular binary encoding technique. In the Onehot-GAT model, each amino acid was represented by a 20-D feature vector since 20 standard amino acids were considered in the datasets. Each amino acid was positionally assigned to 1 and to encode an amino acid, the assigned position is set to 1, while all the others are set to 0.

# **Word2vec**

Word2vec is one of the word embedding techniques applied in the field of natural language processing. In this work, skip-gram model of word2vec was used to get amino acid embeddings. Skip-gram model was used to predict the surrounding context words for a given target word within a specific window size, meaning that it captured the contextual relationships between words (Mikolov *et al.* 2013). Gensim library of Python was used to create word2vec embeddings for amino acids.

# **t-SNE**

t-SNE (t-distributed stochastic neighbor embedding) is a popular statistical method. It can map data from high-dimensional space to low-dimensional space while preserving the local properties of the dataset. t-SNE is primarily used for dimensionality reduction and is particularly well-suited for the visualization of high-dimensional data. Python library scikit-learn was utilized to perform t-SNE.

# **Model implementation and computational costs**

PGAT-ABPp model was implemented using TensorFlow 2.13.1 (https://www.tensorflow.org/), Keras 2.13.1 (https://keras.io/), and Python 3.8 (https://www.python.org/). All models were trained using two Nvidia RTX 4080 GPUs.

| PGAT-ABPp | hyperparameters | optimal value |
| --- | --- | --- |
| graph attention stage | activation function  function | LeakyReLU |
| multi-head graph attention stage | number of heads | 6 |
|  | hidden units | 10 |
|  | number of layers | 1 |
|  | activation function | ReLU |
| training | batch size | 32 |
|  | epoch | 500 |
|  | learning rate | 1e-4 |
|  | patience (early stopping) | 50 |
|  | optimizer | Adam |
|  | random seed | 42 |
|  | train data size : validation data size : test data size | 8:1:1 |
|  | total params | 244233 |

Using the sequence ‘AQWFAIQHISLNPPRSTIAMRAINNYRWR’ (ABP) as an example input (also referenced in comparison table below), the structure prediction section takes about 3 minutes using ColabFold (T4 GPU allocated by Google Colaboratory), while the data preparation and final identification section takes about 3.90 seconds. The output layer gives a result of 0.9995, which means that PGAT-ABPp recognizes this sequence as an ABP. Note that the time required for structure prediction varies with the length and complexity of the sequence.

For those interested in model training, it is important to note that additional structure prediction time is required, which depends on the sequence amount and complexity in the dataset. We made a time consumption comparison of PGAT-ABPp with other methods. Results show that the training and prediction times for PGAT-ABPp (excluding structure prediction) are comparable to those of other methods. Note: this comparison is not rigorous and is provided merely as an example to demonstrate that PGAT-ABPp does not require an excessive amount of time to train.

| Methods | Training time (s) | Prediction time (s) |
| --- | --- | --- |
| AMPDLMD | 56.58 | 1.42 |
| UniDL4BioPep | 568.83 | 5.99 |
| sAMPpred-GAT | 73.00 | 10.65 |
| AMPpred-MFA | 107.08 | 8.80 |
| PGAT-ABPp | 119.23 | 3.90 |

Note: the time required for structural prediction is not included for sAMPpred-GAT and PGAT-ABPp.

# **References**

Mirdita M, Schütze K, Moriwaki Y, *et al.* ColabFold: making protein folding accessible to all. *Nat Methods* 2022;**19**:679-82.

Elnaggar A, Heinzinger M, Dallago C, *et al.* ProtTrans: towards cracking the language of lifes code through self-supervised deep learning and high performance computing. *IEEE Trans Pattern Anal Mach Intel* 2021;**44**:7112-27.

Janczyk M, Pfister R. One-Way Analysis of Variance (ANOVA). In: *Understanding Inferential Statistics.* Berlin, Heidelberg: Springer, 2023, 97-125.

Mikolov T, Chen K, Corrado G, *et al.* Efficient estimation of word representations in vector space. *arXiv Preprint*, arXiv:1301.3781 2013.

Supplementary Figures and Tables


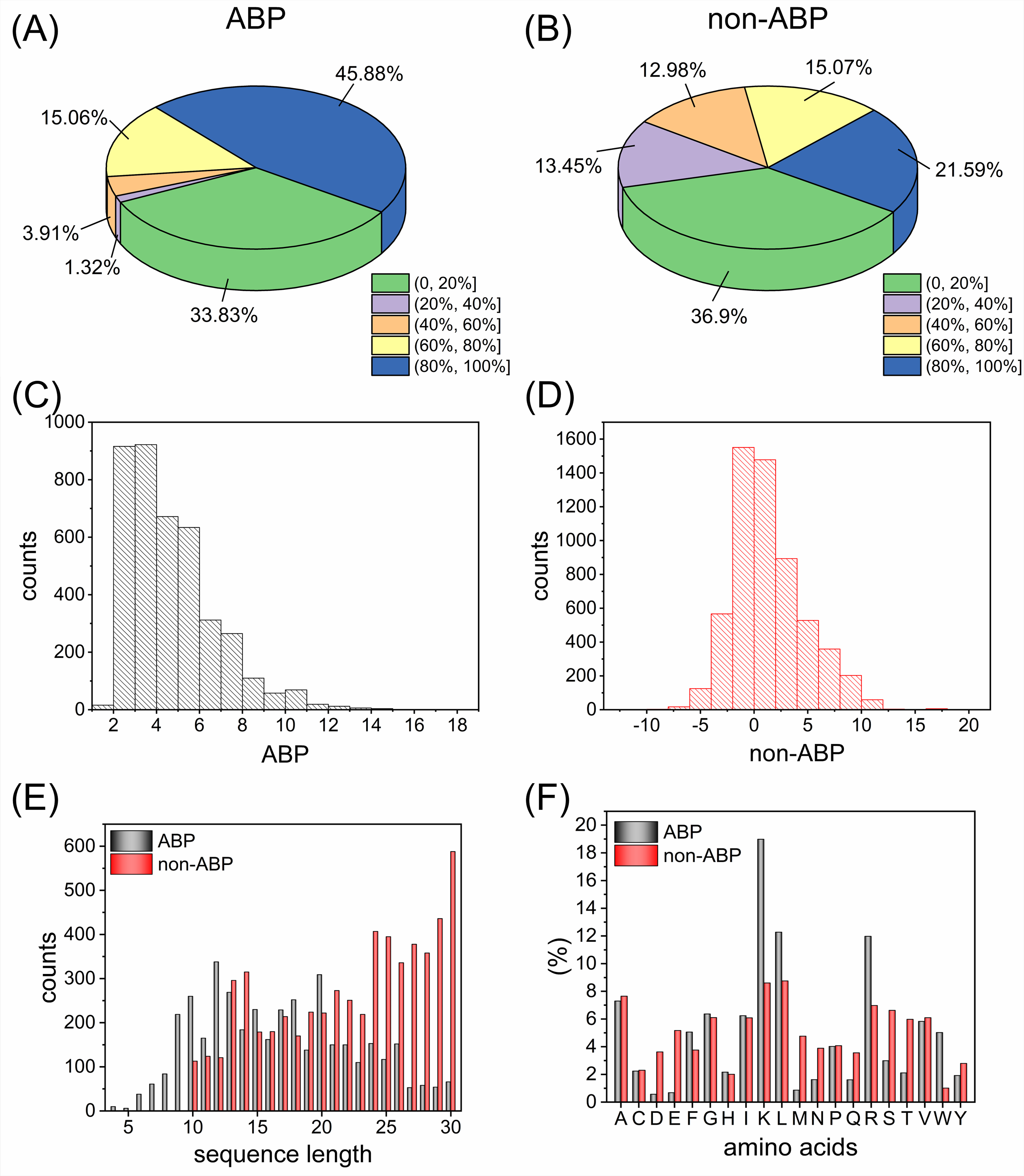


# **Figure S1.** Statistics of the independent test dataset. Proportions of peptides with different α-helix contents within (A) ABPs and (B) non-ABPs. Different colors represent different α-helix content ranges, and the percentage of each sector in the pie chart represents the proportion of peptides within the corresponding range relative to all the peptides being analyzed. Charge distribution histogram of (C) ABPs and (D)non-ABPs. (E) Sequence lengths distribution of ABPs and non-ABPs. (F) Distribution of amino acids in ABPs and non-ABPs.





**Figure S2.** Performance of PGAT-ABPp with different threshold distances (*D_th_*) on the independent test dataset. The cutoff distance to truncate van der Waals interactions is typically set between 8 Å and 12 Å, while 4 Å is often considered a rough distance threshold for hydrogen bonds. Given these considerations, we chose 6 Å-20 Å as the range to examine the threshold distance for the contact map. Using the distance between C_α_-C_α_ atoms as a rough measure of residue connection allows for the use of a large step size. Therefore, a step size of 2 Å was selected to test the influence of threshold distance on the metrics of the model. It can be seen that PGAT-ABPp performed best on the test dataset across all the metrics when *D_th_* was set to 10 Å (green line with upper triangle symbol), the optimal *D_th_* was determined to be 10 Å.


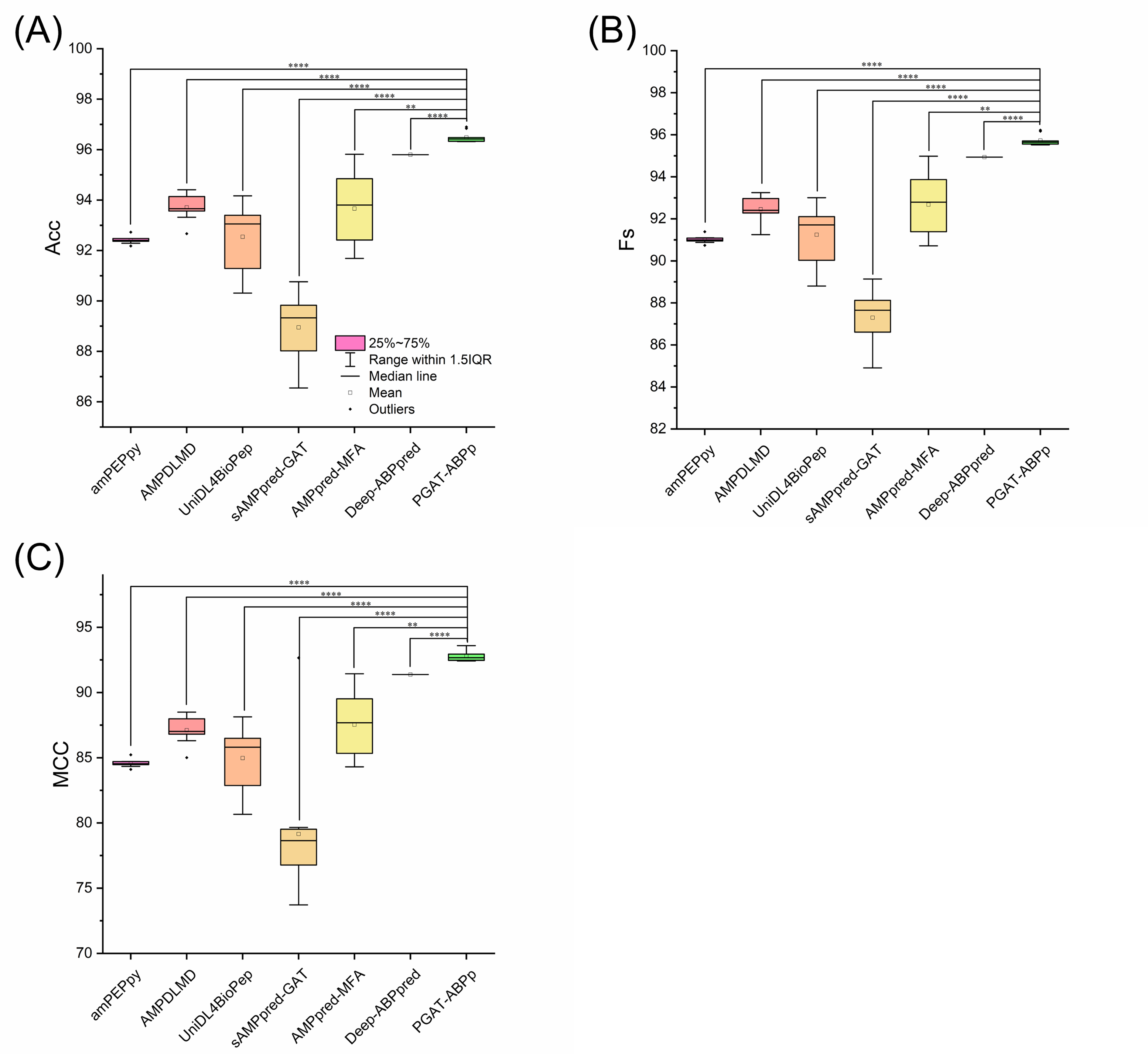


**Figure S3.** (A) Acc, (B) Fs and (C) MCC metrics of amPEPpy, AMPDLMD, UniDL4BioPep, sAMPpred-GAT, AMPpred-MFA, Deep-ABPpred and PGAT-ABPp (mean ± s.d.). Statistical analysis was conducted using one-way ANOVA, Tamhane's T2 test was chosen for post-hoc analyses. ns means *p* > 0.05 (no ), * means *p* < 0.05, ** means *p* < 0.01, *** means *p* < 0.001 and **** means *p* < 0.0001.

**
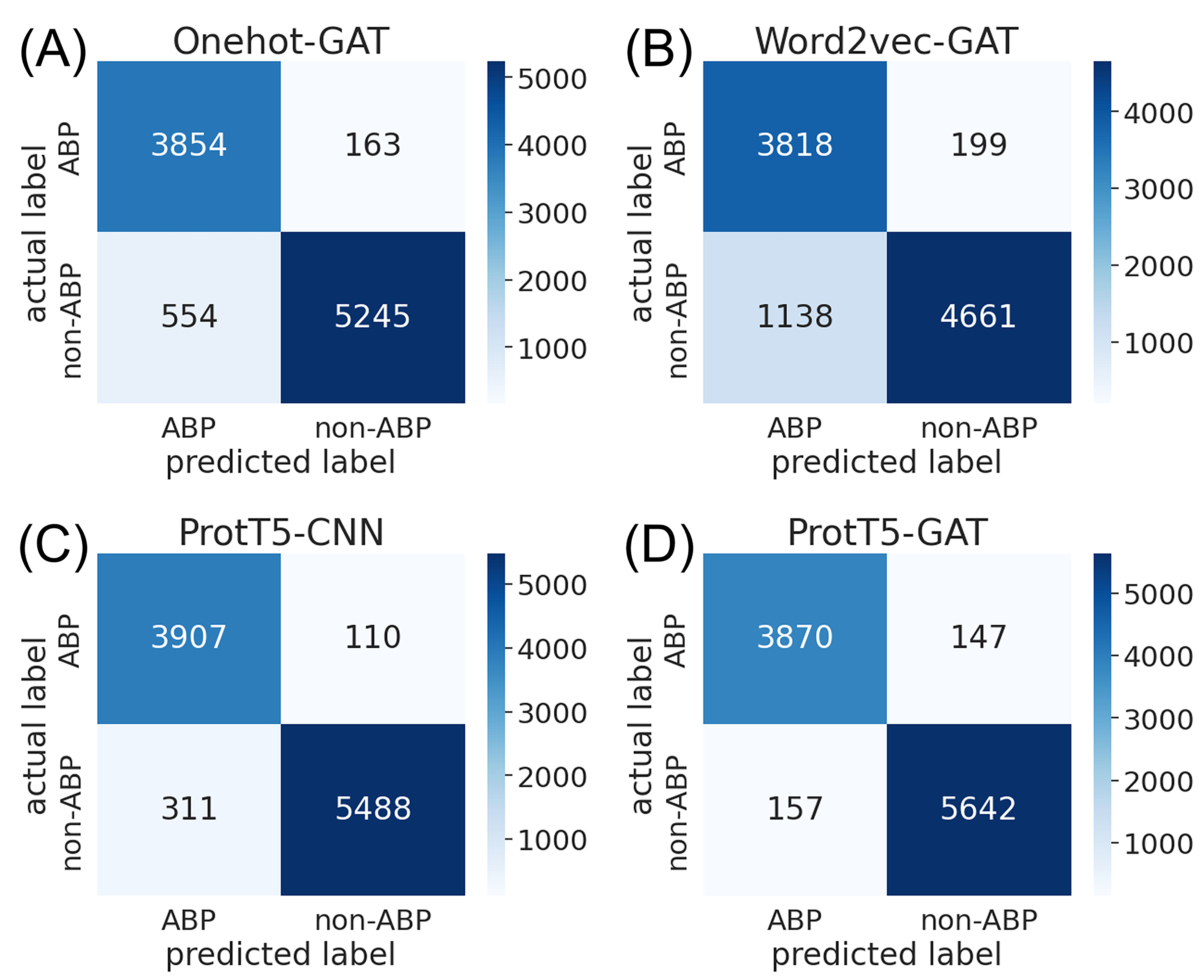
**

# **Figure S4.** Confusion matrix plots on the independent test dataset for (A) Onehot-GAT, (B) Word2vec-GAT, (C) ProtT5-CNN, and (D) ProtT5-GAT (our model).

**Table S1.** Performance of PGAT-ABPp with different graph attention layers on the independent test dataset

| Layers | Acc (%) | Pr (%) | Sp (%) | AUC | Fs | MCC |
| --- | --- | --- | --- | --- | --- | --- |
| 1 | **96.90** | 96.10 | 97.29 | **0.9949** | **0.9622** | **0.9360** |
| 2 | 96.38 | **96.88** | **97.90** | 0.9930 | 0.9552 | 0.9251 |
| 3 | 95.81 | 95.12 | 96.64 | 0.9904 | 0.9487 | 0.9133 |
| 4 | 96.33 | 96.35 | 97.52 | 0.9938 | 0.9548 | 0.9240 |
| 5 | 95.01 | 91.48 | 93.76 | 0.9908 | 0.9407 | 0.8988 |
| 6 | 94.95 | 90.31 | 92.71 | 0.9869 | 0.9408 | 0.8993 |

Note: Best performance of each metric is marked in bold.
